# RING-finger protein 6 promotes colorectal tumorigenesis by transcriptionally activating SF3B2

**DOI:** 10.1038/s41388-021-01872-9

**Published:** 2021-10-05

**Authors:** Hui Xu, Chi Chun Wong, Weilin Li, Yunfei Zhou, Yan Li, Lifu Wang, Lei Liu, Jun Yu

**Affiliations:** 1 Department of Gastroenterology, Ruijin Hospital, Shanghai Jiaotong University School of Medicine, Shanghai, China; 2 Institute of Digestive Disease and Department of Medicine and Therapeutics, State Key Laboratory of Digestive Disease, Li Ka Shing Institute of Health Sciences, The Chinese University of Hong Kong, Hong Kong, China; 3 Department of Genetics, Beth Israel Deaconess Medical Center, Harvard Medical School, Boston, MA USA

**Keywords:** Chemotherapy, Gastrointestinal cancer, Oncogenes

## Abstract

RNF6 is a RING finger protein with oncogenic potential. In this study, we established colon-specific RNF6 transgenic (tg) mice, and demonstrated that RNF6 overexpression accelerated colorectal carcinogenesis compared to wild-type littermates in a chemically induced colorectal cancer (CRC) model. To understand whether transcriptional activity of RNF6 underlies its oncogenic effect, we performed integrated chromatin immunoprecipitation (ChIP)-sequencing and RNA-sequencing analysis to identify splicing factor 3b subunit 2 (SF3B2) as a potential downstream target of RNF6. RNF6 binds to the SF3B2 promoter and the overexpression of RNF6 activates SF3B2 expression in CRC cells, primary CRC organoids, and RNF6 tg mice. SF3B2 knockout abrogated the tumor promoting effect of RNF6 overexpression, whereas the reexpression of SF3B2 recused cell growth and migration/invasion in RNF6 knockout cells, indicating that SF3B2 is a functional downstream target of RNF6 in CRC. Targeting of RNF6-SF3B2 axis with SF3B2 inhibitor with pladienolide B suppressed the growth of CRC cells with RNF6 overexpression in vitro and in vivo. Moreover, the combination of 5-fluorouracil (5-FU) plus pladienolide B exerted synergistic effects in CRC with high RNF6 expression, leading to tumor regression in xenograft models. These findings indicate that tumor promoting effect of RNF6 is achieved mainly via transcriptional upregulation of SF3B2, and that RNF6-SF3B2 axis is a promising target for CRC therapy.

## Introduction

Colorectal cancer (CRC) is one of the most common malignancies worldwide and the third leading cause of cancer deaths [1]. Incidence and mortality rates of CRC in individuals aged under 50 are rapidly rising [2]. Surgical resection remains to be the primary option for CRC treatment; however, few options are available for patients with distant metastasis. Chemotherapy such as platinum-based drugs and 5-fluorouracil (5-FU) are routinely recommended but their effectiveness is limited by intrinsic drug resistance. Hence, there is an urgent need to develop novel therapeutic strategy for CRC.

CRC is a heterogeneous disease with multiple pathogenic mechanisms, which involves genetic and epigenetic alterations [3–5]. Gene amplification is a hallmark of cancer and is frequently detected in CRC [6]. Previously, we identified amplification of RNF6 by whole genome sequencing in CRC specimens, which correlates with its overexpression. As a RING-domain E3 ubiquitin ligase, several studies have demonstrated that RNF6 promotes tumorigenesis in multiple cancers via ubiquitination and degradation of target proteins [7–11]. Alternatively, RNF6 also functions as a transcription factor to control gene expression and modulate germinal differentiation [12]. However, little is known with regards to the potential role of RNF6-mediated transcriptional regulation in tumor development and progression.

In this study, we have constructed colon-specific *Rnf6* transgenic (tg) mice to assess the role of RNF6 in CRC development. Integrative chromatin immunoprecipitation (ChIP)-sequencing and RNA-sequencing unraveled splicing factor 3b subunit 2 (SF3B2) as a downstream transcriptional target of RNF6. Functional validation showed that RNF6 promotes CRC cell proliferation and invasion in a SF3B2-dependent manner. We also evaluated the therapeutic efficacy of SF3B2 inhibitor, pladienolide B, for the treatment of RNF6-expressing CRC in in vitro and in vivo models.

## Materials and Methods

### Cell lines and CRC patient-derived organoid (PDO) model

The human CRC cell lines (DLD1, HCT116, HT29, and SW480) were purchased from the American Type Culture Collection (ATCC; Manassas, VA) and maintained according to the instructions from ATCC. 293T cell line was purchased from Invitrogen (Thermo Fisher Scientific, Waltham, MA). All cell lines were obtained between 2013 and 2015. Primary CRC organoids (PDO74 and PDO828) were kindly provided by Prof. Catherine O’Brien (Department of Surgery at University Health Network, Canada) and cultured as described previously [13]. All cell lines were free of mycoplasma contamination.

### Generation of Knockout cell line with CRISPR/Cas9

Single guide RNA (sgRNA) oligonucleotides (BGI, Beijing, China) targeting RNF6 or SF3B2 (Supplementary Table 1) were cloned into lentiCRISPR v2 (Addgene #52961). Lentivirus was generated by transfection of HEK-293T cells with lentiCRISPR v2, or lentiCRISPR v2-sgRNF6/SF3B2 and packaging plasmids psPAX2 (Addgene #12260) and pMD2.G (Addgene #12259) using FuGENE^®^ HD (Promega). Viral particles in the cell culture supernatant were filtered with 0.45 µm filters and added to target cells.

### Chromatin Immunoprecipitation (ChIP) Assay

According to the EZ-ChIP™ protocol (Merck Millipore), SW480 cells were fixed with 1% formaldehyde for cross-linking at room temperature. Glycine was added to the cells to quench unreacted formaldehyde. After sonication, protein–DNA complexes were immunoprecipitated by anti-RNF6 antibody (proteintech) Supplementary Table 2. Normal IgG was used as negative control. Protein–DNA complex was then captured with Protein A/G Magnetic Beads (Merck Millipore). Finally, immunoprecipitated and input DNA were purified with DNA purification kit (Qiagen). Enrichment of downstream gene was examined by real-time PCR and conventional PCR using primers listed in the Supplementary Table 1. Enrichment of RNF6 binding to the SF3B2 promoter was calculated by percent input method. These experiments were repeated three times independently.

### Electrophoretic mobility shift assay (EMSA) and supershift assays

Nuclear protein was extracted from CRC cells and EMSA was conducted by using a biotin-labeled EMSA kit (Viagene Biotech, China), according to the manufacturer’s instruction. The sequence of the wild-type probe is 5′-TCAACTCAAAGTTTCCTCTCC TCCAGG-3′. The sequence of the mutant-type probe is 5′-TCAACTCAAAGAGCGTGCTCCTCCAGG-3′. Briefly, 3 µg of nuclear proteins were incubated with 10×binding buffer, 1.0 µg poly (dI-dC), and 0.5 µL biotin-labeled probes for 20 min at room temperature. Specificity of the DNA protein complex was confirmed by competition with unlabeled cold-competitor oligonucleotide or mutant-type oligonucleotide added to the mixture. In the supershift assays, 0.1 or 0.4 μg of RNF6 antibody (proteintech) was added to the respective reactions and incubated for 30 min at room temperature, with normal rabbit IgG as a control. The reaction mixtures were separated by polyacrylamide gel electrophoresis and transferred to a nylon membrane. Finally, the membrane was incubated with chemiluminescence substrate buffer, and the bands were visualized using CoolImager (Viagene Biotech, China).

### Primary CRC tumor and normal tissue samples

Sixteen paired primary CRC and adjacent non-tumor specimens were obtained from treatment naïve CRC patients at the Prince of Wales Hospital, the Chinese University of Hong Kong. A cohort of 151 primary colorectal tumors and adjacent normal tissue RNA were obtained from Peking University Cancer Hospital (Beijing, China). Samples were confirmed histologically and staged according to TNM staging system. CRC data were also downloaded from the TCGA Colon and Rectal Cancer database (606 patients). The study was approved by the Clinical Research Ethics Committee of The Chinese University of Hong Kong and Peking University Cancer Hospital. All subjects provided informed consent for obtaining the study specimens. This study was carried out in accordance with the Declaration of Helsinki of the World Medical Association.

### Generation of intestinal-specific RNF6-knockin mouse

Conditional *Rnf6* tg mice (pCAG-loxp-stop-loxp-Rosa26-RNF6) were generated by Biocytogen (China). RNF6-IRES (internal ribosomal entrysite)-eGFP (enhanced green fluorescent protein) was cloned into the Rosa26 WT allele to generate a gene-targeting vector, which were then transfected into embryonic stem cells with C57BL/6 background. After clone selection and identification by PCR and southern blot, positive clones were injected into mouse blastocysts to generate chimeric mice. Chimeric mice were mated with WT C57BL/6 mice to obtain the Rosa26-RNF6 mice. CDX2-CreER^T2^ tg mice were purchased from The Jackson Laboratory, which express CreER^T2^ in adult epithelium of the distal intestinal tract under the CDX2 gene promoter. Rosa26-RNF6 mice were crossed to CDX2-CreER^T2^ to generate *Rnf6* tg/CDX2-Cre mice.

### Azoxymethane (AOM)-induced CRC mouse model

To drive the intestinal-specific overexpression of RNF6, 7-week-old *Rnf6* tg/CDX2-Cre mice were intraperitoneally injected with TAM (100 mg/kg in corn oil) for 4 consecutive days. Two weeks after the first dose of TAM, mice were injected intraperitoneally with 10 mg/kg AOM (Sigma-Aldrich) once a week for 6 consecutive weeks [14]. Mice were monitored twice a week and sacrificed 25 weeks after the first AOM dose. Tumor size and multiplicity were measured, and histology evaluation were performed. All experimental protocols were approved by the Animal Ethics Committee of the Army Medical University (Chongqing, China).

### Xenograft studies

Control vector or RNF6 stably overexpressing HCT116 cells (2 × 10^6^ cells/tumor) were injected subcutaneously into the right dorsal flank of 6-week-old male Balb/c nude mice. Six days after implantation, mice were randomly divided into two groups: (1) vehicle and (2) pladienolide B. Pladienolide B (Santa Cruz) (3 mg/kg) was given intraperitoneally to each mouse on day 0, 2, 4, and 6, as described previously [15]. The tumor size was measured every 2 days and the tumor volume was calculated according to the following: tumor volume (mm^3^) = length × (width)^2^/2. For the combination treatment study, mice injected with RNF6-overexpressing cells were randomly divided into four groups when the tumor volume reached ~50 mm^3^, and treated with (1) vehicle, (2) 5-FU, (3) Pladienolide B, and (4) 5-FU + Pladienolide B. Pladienolide B was given as above, and 5-FU (30 mg/kg) was given intraperitoneally once every 5 days. All experimental procedures were approved by the Animal Ethics Committee of the Chinese University of Hong Kong.

### Statistical analysis

All statistical tests were performed using SPSS or GraphPad Software. All data were presented as mean ± SD. The Pearson correlation coefficient was used to evaluate the correlation between SF3B2 and RNF6 expression in clinical samples. Univariate and multivariate Cox regression analysis was performed to assess the prognostic value of SF3B2 expression. Overall survival in relation to SF3B2 expression was evaluated by Kaplan–Meier survival curve and the log-rank test. Mann–Whitney *U-*test or Student’s *t*-test was performed to compare the variables between two groups. Fisher’s exact test was used to evaluate the proportional difference in categorical variables between groups. The difference in cell viability and tumor growth was determined by repeated-measures analysis of variance. Value of *P* < 0.05 was taken as statistical significance.

Other experimental methods are provided in the Supplemental Information

## Results

### Colon-Specific RNF6 tg overexpression in mice aggravates AOM-induced colorectal carcinogenesis

**Fig. 1:**
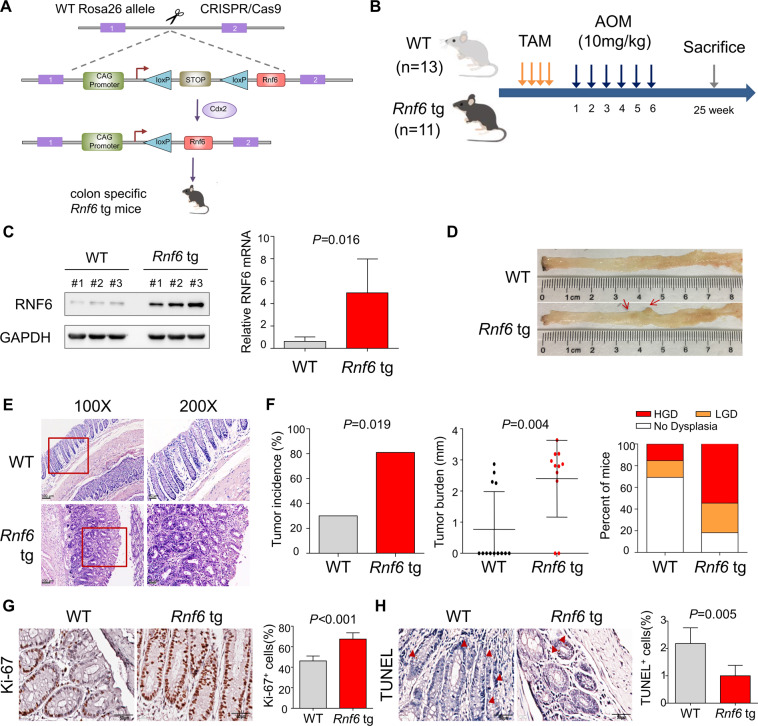
Colon-specific tg RNF6 expression accelerates AOM-induced CRC in mice. **A** Scheme for the generation of mice with colon-specific RNF6 transgenic (tg) expression. **B** Experimental design for AOM-induced CRC in mice. The numbers of wild-type and RNF6 tg mice are 13 and 11, respectively. **C** Western blot and RT-qPCR confirmed overexpression of RNF6 in the colon of RNF6 tg mice. GAPDH was used as a loading control. **D** Representative dissection micrographs of colon tumors from AOM-induced CRC in mice. **E** H&E staining of wild-type and RNF6 tg mouse colons. Colon-specific RNF6 overexpression increased tumor incidence and extent of dysplasia. **F** CRC tumor incidence, tumor burden, and percentage of mice displaying the highest extent of dysplasia in wild-type and RNF6 tg mice. LGD low-grade dysplasia, HGD high-grade dysplasia. **G** Ki-67 staining of cell proliferation in wild-type and RNF6 tg mice. Colon-specific RNF6 overexpression increased cell proliferation. **H** TUNEL analysis of apoptosis in wild-type and RNF6 tg mice. Colon-specific RNF6 overexpression reduced apoptosis. Data were expressed as mean ± SD.

To establish colon-specific RNF6 tg mice, we crossed RNF6 tg mice to CDX2-CreER^T2^ mice, resulting in colon-specific RNF6 overexpression (Fig. 1A). To investigate the functional significance of RNF6 in spontaneous CRC in mice, we utilized a chemically (AOM)-induced model of CRC. After tamoxifen (TAM) at 2 months of age to activate RNF6 tg expression, mice were treated with AOM to induce colorectal tumorigenesis (Fig. 1B). Overexpression of RNF6 mRNA and protein in the colon tissues of RNF6 tg mice was confirmed by Western blot, RT-PCR, and immunohistochemistry analysis (Fig. 1C and Supplementary Fig. 1A). At the end of the study, more RNF6 tg developed macroscopic tumors (9 out of 11) compared to wild-type mice (4 out of 13) (*P* = 0.019) (Fig. 1D). H&E staining showed that RNF6 tg mice displayed high-grade dysplasia at a higher frequency, confirmed CRC formation in RNF6 tg mice (Fig. 1E). Moreover, RNF6 tg mice showed a significant increase in both tumor multiplicity (1.5 vs 0.3; *P* = 0.003) and burden (2.4 vs 0.8 mm; *P* = 0.004) compared to wild-type mice (Fig. 1F and Supplementary Fig. 1B). In addition, we found that wild-type mice from HGD group also expressed higher levels of RNF6 mRNA and protein compared to wild-type mice with no dysplasia in their colon tissues (Supplementary Fig. 1C). We next performed Ki-67 and PCNA staining to evaluate cell proliferation (Fig. 1G and Supplementary Fig. 1D). Compared to WT group, colon tumors derived from *Rnf6* tg mice exhibited higher Ki-67 and PCNA scores, consistent with the pro-tumorigenic effect of RNF6. We also performed TUNEL staining to evaluate apoptosis. Colon tumors derived from RNF6 tg mice had reduced apoptosis compared to WT mice (Fig. 1H). Collectively, our data indicate that RNF6 overexpression aggravates AOM-induced colorectal tumorigenesis in mice.

### Identification of SF3B2 as a transcriptional target for RNF6 by integrated analysis of ChIP-sequencing and RNA-sequencing datasets

**Fig. 2:**
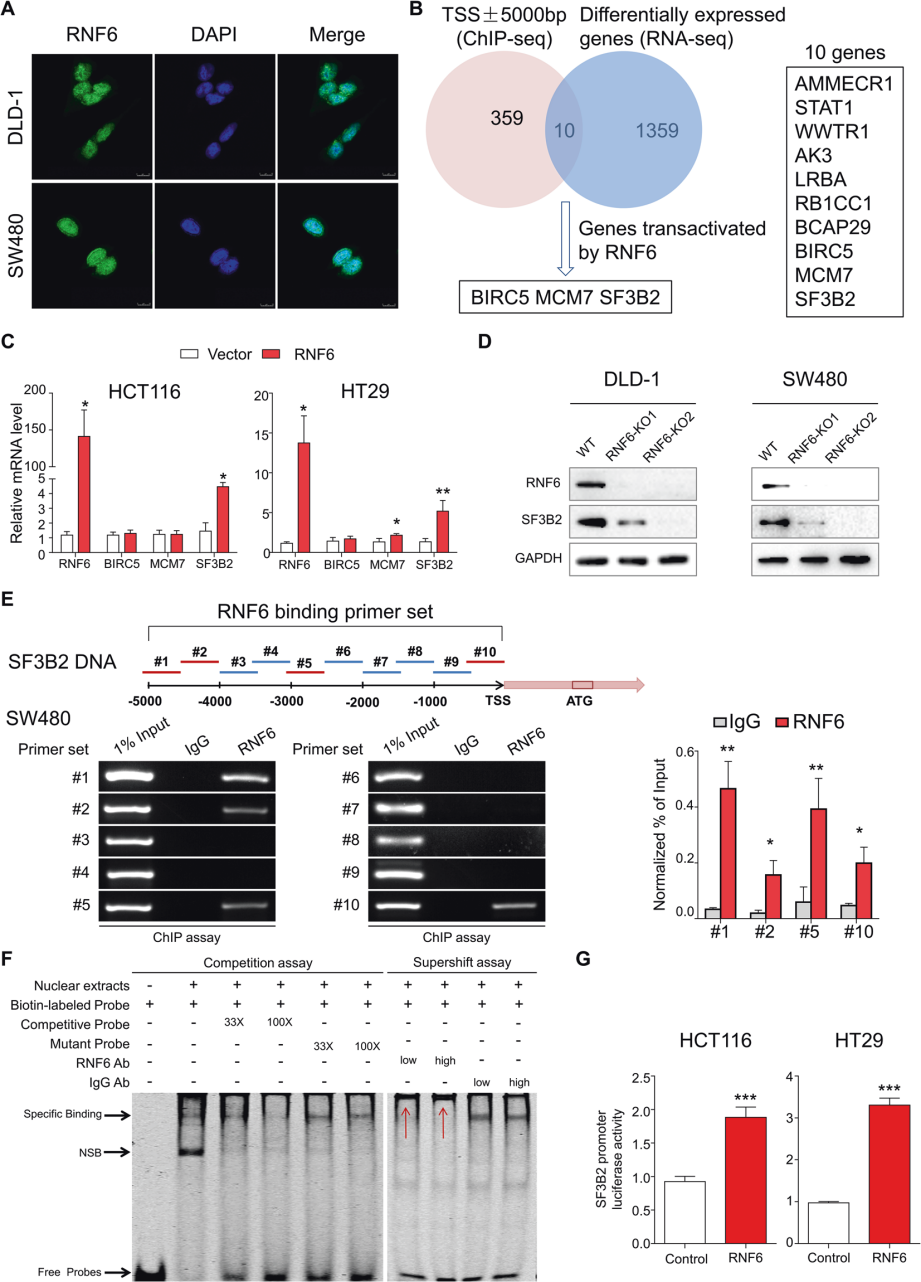
SF3B2 is a transcriptional target of RNF6 via the integrated analysis of ChIP-sequencing and RNA-sequencing. **A** Confocal microscope image of RNF6 localization by immunofluorescence (scale bar 10 μm). **B** Candidate genes were selected based on: (1) ChIP-seq RNF6 binding peaks with 5000 bp of TSS and (2) differential expression (>1.5-fold) in RNA-seq dataset. **C** mRNA expression of candidate genes upon RNF6 overexpression in HCT116 and HT29 by qPCR (*n* = 3, performed in triplicate). Only SF3B2 mRNA was consistently increased in HCT116 and HT29 cells overexpressing RNF6. **D** Expression of SF3B2 upon RNF6 knockout in DLD1 and SW480 cells by Western blot. GAPDH was used as a loading control. **E** Schematic figure summarizing RNF6 ChIP-PCR primer sets in the SF3B2 promoter region (upper). ChIP assay was performed in SW480 cells to confirm RNF6 binding to the SF3B2 promoter (lower left). ChIP-qPCR validation of RNF6 enrichment in SF3B2 promoter (lower right). **F** EMSA performed with biotin-labeled SF3B2 probes and SW480 nuclear extracts. Specific protein–DNA complexes are indicated by arrows. NSB nonspecific binding. Competitive EMSA result demonstrated that nuclear extracts from SW480 cells induced bands shift for SF3B2. In supershift assay, low or high dose of RNF6 antibody and IgG were added to reaction mixture after the reaction of nuclear extract and SF3B2 probes. Supershifts by anti-RNF6 antibody are indicated with the red arrows. **G** RNF6-induced SF3B2 promoter reporter activity, as determined by dual-luciferase reporter assay (*n* = 3, performed in triplicate). Data were expressed as mean ± SD. *P < 0.05, **P < 0.01, ***P < 0.001.

In our previous study, we found that DLD1 and SW480 cells express high level of RNF6 while HCT116 and HT29 cells with low endogenous RNF6 expression [16]. As RNF6 contains a zinc finger domain, it can also function as a transcription factor. Immunofluorescence staining of DLD1 and SW480 cells overexpressing RNF6 showed that RNF6 is localized in both nucleus and cytoplasm, supporting its potential function as a transcription regulator (Fig. 2A). Meanwhile, IHC staining of mouse and human tumor tissues also showed the same result of RNF6 location (Supplementary Fig. 1A) [16]. To identify direct downstream binding targets of RNF6, we next integrated differential expressed genes (DEGs) analysis of RNA-Seq data in RNF6 knockdown SW480 cells and DNA binding profiles of RNF6 in SW480 cells by ChIP-seq. The experimental strategy is shown in Supplementary Fig. 2A, with the following selection criteria for RNA-seq: (1) log2 normalized fold changes <−0.585 or >0.585; (2) adjusted *P* value <0.05. Knockdown of RNF6 in SW480 cells was confirmed by qPCR and Western blot analysis (Supplementary Fig. 2B). DEG profiling identified 1004 upregulated genes and 355 downregulated genes in RNF6 knockdown SW480 cells. KEGG pathway enrichment analyses revealed that these DEGs were enriched actin cytoskeleton, cell cycle, pathways in cancer, p53 signaling pathway, and focal adhesion (Supplementary Fig. 2C). For ChIP-seq, we first mapped RNF6 peaks to gene regulatory features such as promoters, introns, and intergenic regions (Supplementary Fig. 2D). For integrative analysis of DEG data and ChIP-seq, we restricted our search to RNF6 binding sites that lie within 5000 bp of the transcription start site (TSS), which narrowed down the candidates to ten genes (Fig. 2B). By focusing on the genes that were transactivated by RNF6, we finalized the candidates to three—BIRC5, MCM7, and SF3B2. Expression of the other seven candidates was repressed by RNF6 and were not considered further. To validate the three gene candidates, we performed qPCR analysis of their mRNA expression in CRC cells with overexpression/knockdown of RNF6. As shown in Fig. 2C, only SF3B2 mRNA was consistently increased in HCT116 and HT29 cells overexpressing RNF6, whilst being downregulated after RNF6 knockout in DLD1 and SW480 cells (Supplementary Fig. 2E). Western blot also confirmed the downregulation of SF3B2 protein after RNF6 knockout in DLD1 and SW480 cells (Fig. 2D). To examine whether RNF6 might regulate SF3B2 in a posttranscriptional manner, we asked if RNF6 can interact with SF3B2. However, co-immunoprecipitation assays revealed no significant interaction between the two proteins (Supplementary Fig. 2F).

RNF6 binding motif with a core sequence “TTTCCT” was identified from the ChIP-Seq data. We then performed ChIP-PCR assay using ten pairs of primers covering the promoter region of SF3B2 from (−5000 to 0 bp) (Fig. 2E). ChIP-PCR demonstrated that RNF6 binds to the SF3B2 promoter region at no less than four sites (Fig. 2E). Enrichment of SF3B2 promoter by RNF6 pulldown was confirmed by ChIP-qPCR (Fig. 2E). To verify the ChIP results, we next performed electrophoretic mobility shift assay (EMSA) using the promoter region of SF3B2 as the probe. Nuclear extracts expressing RNF6 from SW480 cells induced bands shift for SF3B2 (Fig. 2F). Specificity of SF3B2 promoter was supported by the competitive binding of unlabeled DNA probe to RNF6, which prevented binding of biotin-labeled probes, whereas its binding was not affected by unlabeled mutation probe. The results revealed that SF3B2 could be the direct target of RNF6 in CRC. We next performed supershift assay. The addition of RNF6-specific antibody to the reaction supershifted the probes, but control IgG failed to shift the probes (Fig. 2F), thus confirming the specificity of the RNF6-DNA complex. Moreover, RNF6 expression significantly induced *SF3B2* promoter-driven transcription, as evidenced by luciferase reporter assays (Fig. 2G), thereby confirming that RNF6 activated SF3B2 transcription.

**Fig. 3:**
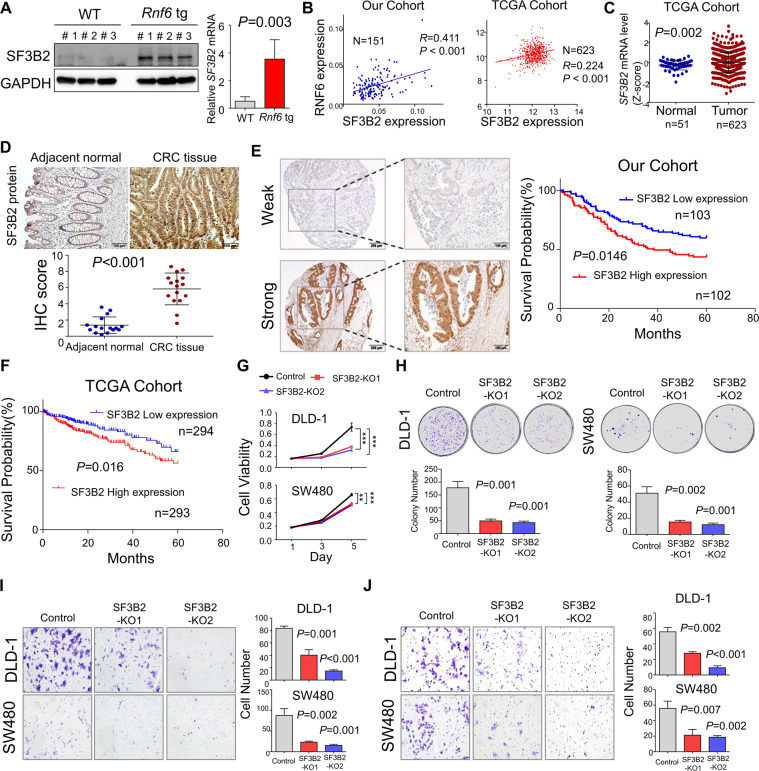
RNF6 upregulates SF3B2, a novel oncogenic factor in CRC. **A** SF3B2 protein and mRNA expression in colon tissues from wild-type and RNF6 tg mice was determined by Western blot and RT-qPCR, respectively. Ectopic expression of RNF6 increased SF3B2 protein and mRNA in the RNF6 tg mice colon. GAPDH was used as a loading control. **B** RNF6 mRNA positively correlated with SF3B2 mRNA in our cohort (*n* = 151) and TCGA dataset (*n* = 623). **C** mRNA expression of SF3B2 in TCGA cohort. SF3B2 mRNA levels were significantly higher in CRC tumor tissues (*n* = 623) compared with normal tissue (*n* = 51) from TCGA cohort. **D** IHC staining of colon tissues for SF3B2 and quantification of IHC staining in normal colon tissue and paired CRC tumors (*n* = 16). **E** SF3B2 protein expression in 205 CRC tissues from our cohort was assessed by IHC staining (left). Kaplan–Meier survival curve of overall survival according to SF3B2 nuclear expression in CRC tissues (right). **F** Kaplan–Meier survival curve of overall survival according to SF3B2 mRNA expression in TCGA cohort. CRC patients with high SF3B2 protein expression had significantly shorter survival. **G** Cell viability was decreased after SF3B2 knockout with sgRNA in DLD1 and SW480 cells (*n* = 3, performed in triplicate). **H** SF3B2 knockout suppressed colony formation in DLD1 and SW480 cells (*n* = 3, performed in triplicate). **I** Effect of SF3B2 knockout on cell migration by transwell assay in DLD1 and SW480 cells (*n* = 3, performed in triplicate). **J** Effect of SF3B2 knockout on cell invasion by Matrigel-coated transwell assay in DLD1 and SW480 cells (*n* = 3, performed in triplicate). Data were expressed as mean ± SD. **P < 0.01. ***P < 0.001.

We next examined the correlation between RNF6 and SF3B2 expression in vivo. In line with our in vitro results, we found that *Rnf6* tg mice had increased SF3B2 protein and mRNA expression in colon tissues as compared to WT mice (Fig. 3A). In line with our findings, SF3B2 mRNA expression also showed a positive correlation with RNF6 mRNA expression in CRC samples from our cohort (*R* = 0.411, *P* < 0.001), TCGA cohort (*R* = 0.224, *P* < 0.001) (Fig. 3B), and in human cancer cell lines encyclopedia (CCLE, https://depmap.org/) (*R* = 0.142, *P* < 0.0001) (Supplementary Fig. 2G), implying a positive association between RNF6 and SF3B2 expression in human CRC.

### SF3B2 exerts an oncogenic effect in CRC

Previous studies indicated that high SF3B2 expression was associated with aggressive phenotypes in prostate cancer [15]. However, the functional significance of SF3B2 in CRC is unknown. We first determined SF3B2 mRNA expression in our CRC cohort. SF3B2 mRNA expression was upregulated in 64.9% (98/151) of primary CRC tissues compared to their adjacent non-tumor tissues (*P* < 0.0001; Supplementary Fig. 3A). Consistently, SF3B2 mRNA was overexpressed in TCGA dataset (Fig. 3C). We also performed IHC for 16 paired colon tumor and adjacent normal tissues. SF3B2 protein expression was significantly increased in colon tumors compared to adjacent non-tumor tissues (Fig. 3D). We then evaluated the prognostic significance of SF3B2 by IHC on tissue microarrays (TMA) consisting of 205 CRC patients. Kaplan–Meier survival analysis demonstrated that CRC patients with high SF3B2 protein expression exhibited significantly shorter survival (*P* = 0.0146, log-rank test) (Fig. 3E). Multivariate Cox regression analysis showed that high SF3B2 expression was an independent predictor of poor survival of CRC patients (RR, 1.903; 95% CI, 1.258–2.879; *P* = 0.002) (Supplementary Table 3). We further validated the prognostic significance of SF3B2 in TCGA cohort (*n* = 587). Kaplan–Meier curve showed that SF3B2 mRNA expression was associated with poor patient survival (*P* = 0.016, log-rank test) (Fig. 3F). SF3B2 expression was an independent prognostic factor in predicting poor survival of CRC patients in TCGA cohort (Supplementary Table 4).

To investigate the functional role of SF3B2 in CRC, we knockout SF3B2 with two sgRNAs in DLD1 and SW480 cells (Supplementary Fig. 3B). Cell growth and colony formation in SF3B2 knockout DLD1 and SW480 cells was reduced compared to empty control-transfected cells (Fig. 3G, H). SF3B2 knockout also inhibited cell migration (Fig. 3I) and cell invasion (Fig. 3J), as determined by transwell assay and Matrigel invasion assay, respectively. Collectively, these findings suggested that SF3B2 promotes cell growth, migration, and invasion of CRC cells.

### RNF6 promotes CRC proliferation and metastasis in a SF3B2-dependent manner

**Fig. 4:**
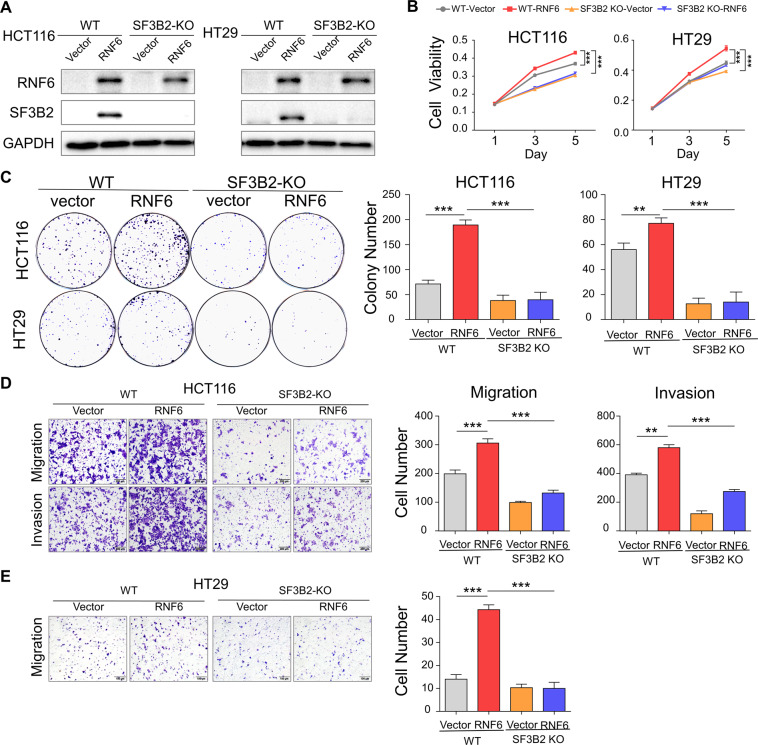
Oncogenic effect of RNF6 in CRC is dependent on SF3B2. **A** SF3B2 knockout and wild-type CRC cells (HCT116 and HT29) were transfected with empty vector or RNF6 overexpression plasmid. RNF6 and SF3B2 protein expression were determined by Western blot. GAPDH was used as a loading control. **B** MTT, **C** colony formation. **D**, **E** Transwell migration and invasion assays (*n* = 3, performed in triplicate) were used to assess tumorigenic functions. These functional assays collectively demonstrated that SF3B2 knockout abolished the effect of RNF6 overexpression on cell proliferation, colony formation, and cell migration and invasion. Data were expressed as mean ± SD. **P < 0.01. ***P < 0.001.

To evaluate whether RNF6 induces tumor progression through regulating SF3B2, we transfected SF3B2 knockout HCT116 and HT29 cells with RNF6 expression plasmid or empty vector. Western blot confirmed RNF6 overexpression in SF3B2 knockout cells (Fig. 4A). Functional assays demonstrated that overexpression of RNF6-promoted cell growth (Fig. 4B), colony formation (Fig. 4C), and cell migration/invasion (Fig. 4D, E) in HCT116 and HT29 cells. However, oncogenic effects of RNF6 were largely abolished when in SF3B2 knockout cells (Fig. 4B–E). Reciprocally, the overexpression of SF3B2 in RNF6 knockout DLD1 and SW480 cells rescued the effect of RNF6 knockout on cell proliferation and colony formation (Supplementary Fig. 4A, B). Taken together, these data suggest that tumor promoting function of RNF6 in CRC was dependent on the upregulation of SF3B2 expression.

### SF3B2 inhibitor pladienolide B suppresses growth of RNF6-overexpressing CRC cells tumors in vitro and in vivo

Recently, several mRNA splicing inhibitors have been developed as antitumor drugs including pladienolide B, which directly bind SF3b complex and its associated splicing processes in cancer. SF3B2 is an integral component of SF3b complex, and pladienolide B was found to exhibit strong antitumor activity and suppress the growth of tumors via repression of spliceosome. Interestingly, alternative splicing analysis of RNA-seq data for SW480-siControl and SW480-siRNF6 cells revealed a significant increase in the number of splicing events associated with intron retention (*N* = 38) after the knockdown of RNF6, consistent with impaired RNA splicing (Supplementary Table 5). We thus hypothesized that pladienolide B could be a potential therapeutic drug for CRC with high RNF6 expression.

**Fig. 5:**
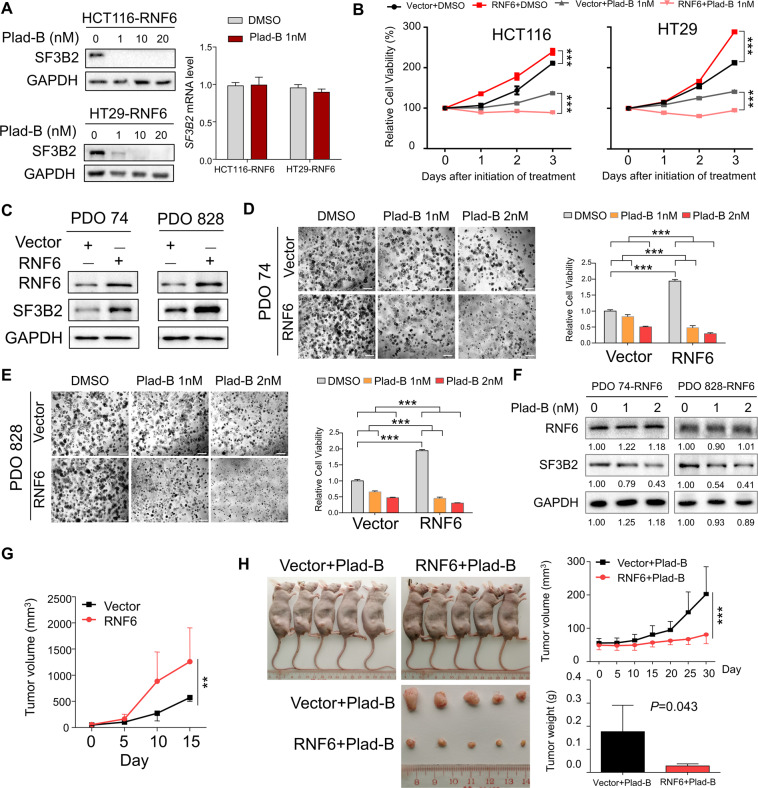
SF3b complex inhibitor pladienolide B suppresses the growth of RNF6-overexpressing tumors in vitro and in vivo. **A** Pladienolide B suppressed SF3B2 protein expression in RNF6-overexpressing HCT116 and HT29 cells as determined by Western blot (left). GAPDH was used as a loading control. Plad-B (1 nM) had no effect on SF3B2 mRNA expression (right). **B** Relative cell viability of vector or RNF6-overexpressing HCT116 and HT29 cells administrated pladienolide B (1 nM) or vehicle (DMSO) (*n* = 3, performed in triplicate). Viability of cells with RNF6 overexpression was reduced compared to vector controls. **C** Two human CRC organoids (PDO74 and PDO828) were successfully transfected with empty vector or RNF6 plasmid. GAPDH was used as a loading control. **D**, **E** Empty vector or RNF6-overexpressing CRC organoids were photographed after treatment with pladienolide B or vehicle (DMSO), and then quantified by cell viability assay (scale bar 200 μm) (*n* = 3, performed in triplicate). The cell viability was normalized to untreated control groups. **F** Pladienolide B (1 or 2 nM) treatment in RNF6-overexpressing PDO74 or PDO828 reduced SF3B2 protein expression, but had no effect on RNF6 protein. GAPDH was used as a loading control. **G** Tumor volume in nude mice bearing HCT116 cells with empty vector or RNF6 overexpression (*n* = 5). **H** Tumor volume and tumor weight in nude mice bearing HCT116 cells with empty vector or RNF6 overexpression were treated with pladienolide B at 3 mg/kg (*n* = 5). Pladienolide B treatment resulted in greater regression in tumors with stable RNF6 overexpression compared with tumors with empty vector. Data were expressed as mean ± SD. **P < 0.01. ***P < 0.001.

To validate the on-target inhibition of SF3B2 by pladienolide B, we first examined its effect on SF3B2 expression in CRC cells. RNF6-overexpressing HCT116 and HT29 cells were treated with different doses of pladienolide B (1 to 20 nM). The results indicated that pladienolide B inhibited SF3B2 protein expression but had no significant effect on SF3B2 mRNA expression (Fig. 5A). We then treated HCT116 and HT29 cells expressing control vector or RNF6 with vehicle or pladienolide B (1 nM). Cell viability assay showed that pladienolide B inhibited the cell proliferation of RNF6-overexpressing cells to a greater extent compared to controls (Fig. 5B). We also investigated the effect of pladienolide B against a panel of CRC cell lines. Compared to DLD1 and SW480, CRC cells with low RNF6 expression were less sensitive to the growth inhibitory effects of pladienolide B (Supplementary Table 6), consistent with a role of SF3B2 in mediating the oncogenic effect of RNF6.

Next, we tested the effect of pladienolide B in primary CRC-derived organoids. Two human CRC organoids, PDO74 and PDO828, cultured in 3D Matrigel were transfected with empty vector or RNF6 plasmid and treated with pladienolide B. Overexpression of RNF6 also activates SF3B2 expression in primary CRC organoids (Fig. 5C). We found that pladienolide B more effectively suppressed growth in PDO74 and PDO828 organoids overexpressing RNF6 compared to empty vector controls (Fig. 5D, E). Western blot confirmed that pladienolide B suppressed SF3B2 protein expression in PDO74 and PDO828 organoids, while having no effect on RNF6 (Fig. 5F).

To further validate the inhibitory effect of pladienolide B on RNF6-expressing tumors, we administered the drug into nude mice harboring HCT116 xenografts with vector or RNF6 overexpression. In line with our previous data, RNF6 overexpression accelerated tumor growth in the group without pladienolide B treatment (Fig. 5G). In contrast, Pladienolide B-treated, RNF6-expressing HCT116 xenografts have attenuated growth as compared to Pladienolide B-treated HCT116-vector xenografts (Fig. 5H). These data collectively imply that high RNF6 expression contributes to the sensitivity of CRC tumors to pladienolide B.

### Pladienolide B is synergistic with 5-FU in suppressing CRC cells with high RNF6 expression

**Fig. 6:**
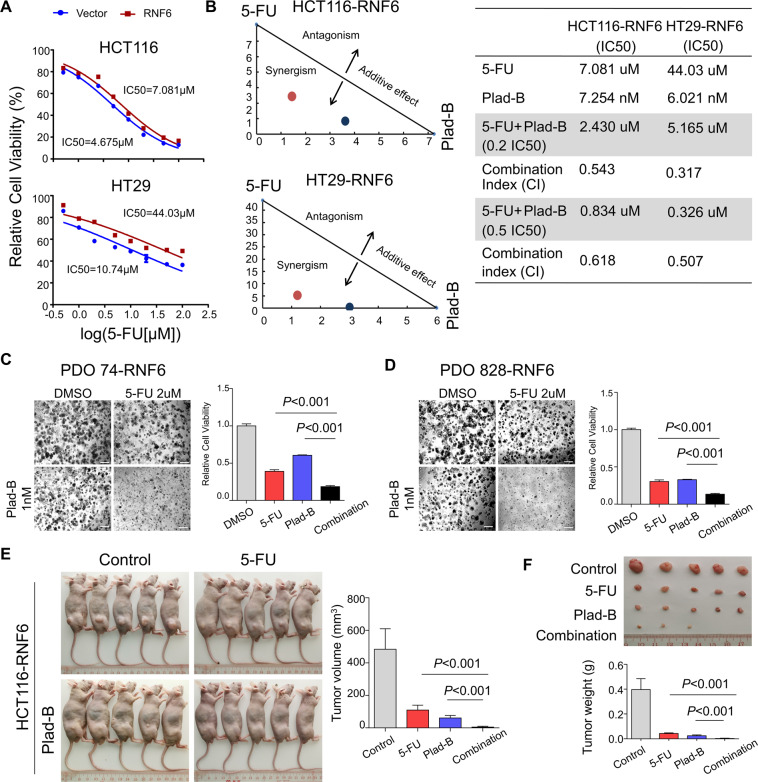
Pladienolide B demonstrates synergistic effects with 5-FU in CRC with high RNF6 expression. **A** Dose-response curve of 5-FU in empty vector or RNF6-expressing HCT116 and HT29 cells. Cells were treated with 5-FU for 72 h. IC_50_ values were significantly increased in cells with RNF6 overexpression compared with the control group. **B** Isobologram analysis of combined effect of 5-FU and pladienolide B (left). RNF6-overexpressing HCT116 and HT29 cells were treated with 5-FU, pladienolide B, or their combination as indicated to determine the interaction of drugs. IC_50_ and confidence interval (CI) values were shown in the right table. CI values were calculated for quantitative definition of synergism (CI < 1), additive effect (CI = 1) and antagonism (CI > 1). **C**, **D** RNF6-overexpressing CRC organoids (PDO74 and PDO828) were photographed after treatment with 5-FU, pladienolide B, or their combination (scale bar 200 μm). Cell viability was normalized to the untreated control groups (*n* = 3, performed in triplicate). The results showed that proliferation of PDO74 and PDO828 was significantly decreased by combination treatment. **E**, **F** Pladienolide B synergized with 5-FU to suppress the growth of HCT116-RNF6 xenografts in nude mice (*n* = 5). Tumor volume and tumor weight in nude mice bearing HCT116-RNF6 xenografts and given control, 5-FU, pladienolide B, or their combination are shown in **(E)** and **(F)**. Data were expressed as mean ± SD.

Conventional cytotoxic chemotherapy including 5-FU and doxorubicin are widely used in the treatment of cancers. RNF6 functions as an oncogene in CRC; however, whether it plays a role in chemotherapy resistance is unknown. We thus evaluated the effect of RNF6 overexpression on chemotherapeutic efficacy. In HT29 cells, 72 h-IC_50_ values for doxorubicin and 5-FU were 300 nM and 10 μM, respectively (Supplementary Fig. 5A, B). We then treated HT29 control and RNF6-overexpressing cells with 300 nM doxorubicin or 10 μM 5-FU. Cell viability assays revealed that RNF6-overexpressing cells exhibited higher resistance to both doxorubicin and 5-FU (Supplementary Fig. 5A, B). Since 5-FU is commonly used for CRC chemotherapy, we validated our observations by performing dose-response curves of 5-FU in HT29 and HCT116 cells with RNF6 overexpression, which showed that RNF6 significantly increased 72 h-IC_50_ of 5-FU in both cell lines (Fig. 6A). These results indicate that RNF6 mediates 5-FU resistance in CRC cells.

Given that RNF6 confers drug resistance towards 5-FU while concomitantly promoting sensitivity to pladienolide B, we reasoned that the combination of 5-FU plus pladienolide B could be a promising strategy for targeting CRC with high RNF6 expression. We first quantitatively examined synergism between 5-FU and pladienolide in vitro according to the Chou–Talalay Method [17]. We found that the combination of 5-FU and pladienolide was highly synergistic in both HCT116-RNF6 and HT29-RNF6 cells, as evidenced by the combination index (CI) value <1 (Fig. 6B). Next, we sought to test the synergistic effect of pladienolide B and 5-FU in CRC organoids. In PDO74 and PDO828 organoids overexpressing RNF6, 5-FU plus pladienolide B suppressed viability to a greater extent as compared to single drug treatment (Fig. 6C, D). To validate the synergistic effect in vivo, we injected HCT116-RNF6 cells into nude mice. When tumors reached ~50 mm^3^, mice were randomized into four groups as follows: PBS, 5-FU, pladienolide B, and 5-FU + pladienolide B. As shown in Fig. 6E, F, 5-FU plus pladienolide B synergistically induced the tumor regression of HCT116-RNF6 xenografts compared to control or single drug treatment in vivo, both in terms of tumor weight (*P* < 0.001) and volume (*P* < 0.001). Taken together, these data suggest that the combination of 5-FU and pladienolide B may be a novel strategy in treating CRC with high RNF6 expression.

## Discussion

RNF6, a RING finger E3 ligase, has been shown to be an oncogene by virtue of its E3 ligase activity [7–11, 18, 19]. In this study, we have established a colon-specific RNF6 tg mice model, and demonstrated that RNF6 overexpression accelerated AOM-induced spontaneous CRC in vivo. Compared to wild-type mice, RNF6 tg mice have increased cell proliferation and suppressed apoptosis, consistent its pro-tumorigenic phenotype. These findings are largely in line with our previous observations in CRC cell lines [7]. Our results provide compelling evidence that RNF6 overexpression aggravates CRC progression.

The majority of RNF proteins are believed to function as E3 ubiquitin ligases, however, their RING domains can also bind to DNA and mediate transcription. Here, we are the first to demonstrate that RNF6 could also function as a transcription factor in promoting oncogenesis. Our integrative analyses of ChIP-seq and RNA-seq datasets identified SF3B2 as a key downstream target of RNF6 in CRC. We showed that RNF6 binds to the promoter region of SF3B2 to activate its transcription in CRC cells. Moreover, enhanced SF3B2 expression was found in colon tissues of *Rnf6* tg mice. Transcriptional activity of RNF6 appears to be distinct from its E3 ligase activity, as its ligase targets, such as TLE3 and SHP-1 [7, 8], were not identified by our integrative ChIP-seq/RNA-seq analyses. Collectively, we identified a novel mechanism whereby RNF6 promotes transcription activation of SF3B2.

Critically, the oncogenic effect of RNF6 is highly dependent on SF3B2, as the deletion of SF3B2 abrogated RNF6-induced cell growth, colony formation, and cell migration and invasion. Moreover, the overexpression of SF3B2 promoted CRC cell growth and restored malignant phenotypes in cells with RNF6 depletion. These results collectively suggested that SF3B2 functions as a downstream factor essential for RNF6-promoted colorectal tumorigenesis. Besides RNF6, another RNF protein, RNF2, is also reported to possess dual roles in the regulation of oncogenic gene networks via transcriptional upregulation of Cyclin D2 in melanoma [20]. Taken together, we have revealed a novel RNF6-SF3B2 signaling axis that promotes malignant phenotypes in CRC.

SF3B2 is a component of splicing factor SF3B complex involved in pre-mRNA splicing [21]. RNA splicing factors have been implicated in cancers in pro- or anti-tumorigenic activities. In our study, we revealed that SF3B2 was consistently overexpressed in CRC compared to adjacent normal tissues in independent patient cohorts. High expression of SF3B2 is correlated with poor overall survival and is an independent prognostic factor in CRC in our cohort and TCGA dataset. Corroborating these results, SF3B2 knockout in CRC cells suppressed multiple malignant phenotypes including cell growth, migration, and invasion. In agreement with our results, SF3B2 conferred an aggressive phenotype in prostate cancer and was associated with poor overall survival and progression-free survival in many cancers, such as bladder, lung, and breast cancers [15]. However, further investigation will be needed to elucidate the effect of RNF6 on SF3B2-mediated RNA splicing in CRC.

RNA splicing inhibitors are an emerging class of antitumor drugs attempting to reverse the aberrant RNA splicing activities in cancer cells. Pladienolide B, a naturally existing antitumor macrolide, directly binds to spliceosome-associated 130 (SAP130) subunit of the SF3b complex (SF3B1–7), thereby blocking the spliceosome [22, 23]. Notably, pladienolide B suppressed the interaction of SF3B2 with the rest of the SF3b complex [15, 24]. Given that RNF6 inhibitors are not available, we proposed that pladienolide B might be a candidate drug for targeting of RNF6-SF3B2 axis in CRC. As expected, in CRC cell lines and CRC organoids in vitro as well as subcutaneous xenograft models in vivo, pladienolide B demonstrated strong and selective antiproliferative activities towards CRC with high RNF6 expression, confirming that pharmacological blockade of RNF6-SF3B2 axis is a potential approach for suppressing CRC progression.

5-FU-based chemotherapy is the mainstays for the treatment of advanced CRC patients, however, its efficacy is frequently compromised by drug resistance. Here, we revealed the novel association between RNF6 expression and chemotherapy resistance, as CRC cells with ectopic RNF6 expression are more resistant to 5-FU treatment. Against this backdrop, we next tested if pladienolide B could be employed to sensitize high RNF6-expressing CRC cells to 5-FU treatment. Indeed, we found a striking synergistic effect between pladienolide B and 5-FU in CRC with RNF6 overexpression in both in vitro and in vivo models. This work collectively highlights the potential of pladienolide B as a drug candidate, and provides a rationale of combination of 5-FU plus pladienolide B for CRC patients with high RNF6 expression. Apart from pladienolide B, several SF3b inhibitors are currently under preclinical and clinical developments. One such example is E7107, a derivative of pladienolides. E7107 showed inhibition of solid tumor growth with response rate of ~30%; however, it is associated with vision loss in a few patients in clinical trials [25, 26]. More clinical testing will be essential to evaluate the feasibility of targeting SF3b in conjunction with chemotherapy for the treatment of CRC.

**Fig. 7:**
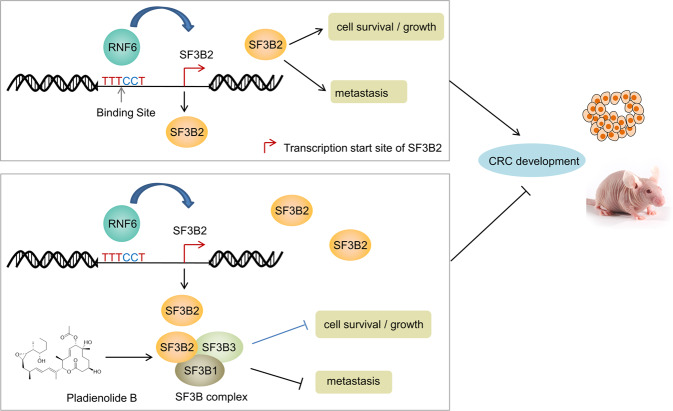
Proposed mechanistic scheme of RNF6-SF3B2 axis in CRC. Our study showed that RNF6 transcriptionally upregulates SF3B2 expression via direct binding to the SF3B2 promoter. Downstream of RNF6, SF3B2 functions as an oncogene that mediates colorectal tumorigenesis. Pladienolide B, an inhibitor of the SF3b complex, is strongly effective in inhibition of tumor growth in CRC with RNF6 overexpression.

In conclusion, RNF6 plays a pro-tumorigenic role in CRC via upregulation of SF3B2. RNF6 binds to promoter and drives the transcription of SF3B2, which in turn, mediates tumorigenic phenotypes in CRC (Fig. 7). The targeting of RNF6-SF3B2 axis with pladienolide B strongly inhibited the growth of RNF6-expressing CRC cells, especially in conjunction with 5-FU. Our findings provide the rationale for further development of therapeutic strategies to attenuate RNF6-SF3B2-mediated CRC.

## Supplementary information

Supplementary Tables

Supplementary Methods

Supplementary Figures
