# Supplementary material for: RING-finger protein 6 promotes colorectal tumorigenesis by transcriptionally activating SF3B2
Source: Oncogene. 2021 Oct 5;40(47):6513–26. doi: 10.1038/s41388-021-01872-9 (PMC8616760; doi:10.1038/s41388-021-01872-9)
Supplement: Supplementary file 1 — Supplementary Tables [file 41388_2021_1872_MOESM1_ESM.docx]

**Supplementary Table 1.** Primers used in this study

| **Primer name** | **Purpose** | **Species** | **Sequence** |
| --- | --- | --- | --- |
| RNF6-CHIP-P1-F | CHIP PCR | Human | CTGCCTGCTCCTAACTCGTC |
| RNF6-CHIP-P1-R | CHIP PCR | Human | CGAGGAGCTCGGTTTCTCTG |
| RNF6-CHIP-P2-F | CHIP PCR | Human | ACTGGTTTCCCCAACCTTGC |
| RNF6-CHIP-P2-R | CHIP PCR | Human | CCTGCAGAGTCAGCTGATGG |
| RNF6-CHIP-P3-F | CHIP PCR | Human | TCACCTTTTCTGGGAGCCCT |
| RNF6-CHIP-P3-R | CHIP PCR | Human | CTGTGCCCTTGGTTACCCTC |
| RNF6-CHIP-P4-F | CHIP PCR | Human | CAGTCTGAGGCCTGCTTAGG |
| RNF6-CHIP-P4-R | CHIP PCR | Human | TGCTCAGAAATGGACCAGGC |
| RNF6-CHIP-P5-F | CHIP PCR | Human | CCCCTTACACCTCCCATTCTG |
| RNF6-CHIP-P5-R | CHIP PCR | Human | CCCCATCTCCAAAACAGGCA |
| RNF6-CHIP-P6-F | CHIP PCR | Human | ACAATCTACCTCACGCTGCT |
| RNF6-CHIP-P6-R | CHIP PCR | Human | GGCACCCCTTCCTCTAAGTG |
| RNF6-CHIP-P7-F | CHIP PCR | Human | CCGCTATCTCTGGACCCTTG |
| RNF6-CHIP-P7-R | CHIP PCR | Human | GCCACCTGATCCCATCCAG |
| RNF6-CHIP-P8-F | CHIP PCR | Human | CCGTGAACAGTCTCTGGGTG |
| RNF6-CHIP-P8-R | CHIP PCR | Human | CCTCAGTTTCCCTGCCTGAG |
| RNF6-CHIP-P9-F | CHIP PCR | Human | ACCAGGCATCAAAGAACCCA |
| RNF6-CHIP-P9-R | CHIP PCR | Human | ACAGCTGGAAGCAATAGGGC |
| RNF6-CHIP-P10-F | CHIP PCR | Human | CTGGATACGCCCTTCTAGGC |
| RNF6-CHIP-P10-R | CHIP PCR | Human | TCCTCCTCCCCCACGATATT |
| β-ACTIN mRNA-F | RT-qPCR | Human | CCCTGGAGAAGAGCTACGAG |
| β-ACTIN mRNA-R | RT-qPCR | Human | AGGTAGTTTCGTGGATGCCA |
| RNF6 mRNA-F | RT-qPCR | Human | TCAGCCTGACTTGAGAGATGG |
| RNF6 mRNA-R | RT-qPCR | Human | TTCGAGTTGCATTTCCTGTG |
| BIRC5 mRNA-F | RT-qPCR | Human | AGGACCACCGCATCTCTACAT |
| BIRC5 mRNA-R | RT-qPCR | Human | AAGTCTGGCTCGTTCTCAGTG |
| MCM7 mRNA-F | RT-qPCR | Human | CCTACCAGCCGATCCAGTCT |
| MCM7 mRNA-R | RT-qPCR | Human | CCTCCTGAGCGGTTGGTTT |
| SF3B2 mRNA-F | RT-qPCR | Human | ACCAAGACTGAGGAAGAAGAGAT |
| SF3B2 mRNA-R | RT-qPCR | Human | TCCAGCAGGCACTGATGA |
| β-ACTIN mRNA-F | RT-qPCR | Mouse | CATTGCTGACAGGATGCAGAAGG |
| β-ACTIN mRNA-R | RT-qPCR | Mouse | TGCTGGAAGGTGGACAGTGAGG |
| RNF6 mRNA-F | RT-qPCR | Mouse | GAAGATGGCAGCAAGAACGTC |
| RNF6 mRNA-R | RT-qPCR | Mouse | AGGGGTGCCTAAAAGATTATGGT |
| SF3B2 mRNA-F | RT-qPCR | Mouse | CGCCTGAGGAATTGGAGTTGGAC |
| SF3B2 mRNA-R | RT-qPCR | Mouse | CTTCTCTACTTGTGCCTGCTGCTC |
| RNF6 gRNA-F1 | CRISPR | Human | CACCGCCATAACAGTTCCTCTTCGT |
| RNF6 gRNA-R1 | CRISPR | Human | AAACACGAAGAGGAACTGTTATGGC |
| RNF6 gRNA-F2 | CRISPR | Human | CACCGTTCGTTCCATCTCTCAAGTC |
| RNF6 gRNA-R2 | CRISPR | Human | AAACGACTTGAGAGATGGAACGAAC |
| SF3B2 gRNA-F1 | CRISPR | Human | CACCGAACAGATGCTCGCTCGTCCC |
| SF3B2 gRNA-R1 | CRISPR | Human | AAACGGGACGAGCGAGCATCTGTTC |
| SF3B2 gRNA-F2 | CRISPR | Human | CACCGCCGAATGAACCGCTTCACTG |
| SF3B2 gRNA-R2 | CRISPR | Human | AAACAGTGAAGCGGTTCATTCGGC |

**Supplementary Table 2.** Antibodies used in this study

| **Ab name** | **Cat#** | **Company** | **Source** | **Dilution** | **Note** |
| --- | --- | --- | --- | --- | --- |
| RNF6 | PA5-68868 | Invitrogen | Rabbit | 1:100 | IHC |
| RNF6 | 20437-1-AP | Proteintech | Rabbit | 1:80/1:800 | IF/WB |
| SF3B2 | ab56800 | abcam | Mouse | 1:10000 | WB |
| SF3B2 | 10919-1-AP | Proteintech | Rabbit | 1:800 | WB/IHC |
| GAPDH | sc-47724 | Santa Cruz | Mouse | 1:800 | WB |
| Ki67 | ab16667 | abcam | Rabbit | 1:500 | IHC |
| PCNA | 2586 | Cell signaling | Mouse | 1:2000 | IHC |

**Supplementary Table 3.** Univariate and multivariate Cox regression analysis of potential poor prognostic factors in 205 colorectal cancer patients from our Hong Kong cohort

| **Variable** | **Univariate analysis**  **HR (95% CI) *p* value** |  | **Multivariate analysis**  **HR (95% CI) *p* value** |
| --- | --- | --- | --- |
| **Age**  >60  ≤60  **Gender**  Female  Male  **Localization**  Rectum  Colon  **Differentiation**  Low  Moderate/High  **TNM stage**  III/IV  I/II  ***SF3B2***  High  Low | 0.927 0.718  (0.615-1.398)  1.00  0.988 0.952  (0.662-1.474)  1.00  1.016 0.941  (0.668-1.545)  1.00  6.202 0.000  (3.472-11.082)  1.00  3.964 0.019  (1.255-12.516)  1.00  1.641 0.016  (1.098-2.452)  1.00 |  | 0.821 0.391  (0.523-1.289)  1.00  0.912 0.656  (0.608-1.368)  1.00  1.002 0.991  (0.645-1.557)  1.00  6.572 0.000  (3.539-12.207)  1.00  3.917 0.023  (1.209-12.690)  1.00  1.903 0.002  (1.258-2.879)  1.00 |

The TNM Staging System is based on the extent of the tumor (T), the extent of spread

to the lymph nodes (N), and the presence of metastasis (M). CI, confidence interval;

HR, hazard ratio. HRs and their 95% CIs were computed by using Cox hazard

proportional regression model.

**Supplementary Table 4.** Univariate and multivariate Cox regression analysis of potential poor prognostic factors in 587 colorectal cancer patients from TCGA cohort

| **Variable** | **Univariate analysis**  **HR (95% CI) *p* value** |  | **Multivariate analysis**  **HR (95% CI) *p* value** |
| --- | --- | --- | --- |
| **Age**  >60  ≤60  **Gender**  Female  Male  **Localization**  Rectum  Colon  **MSI status**  MSS/MSI-L  MSI-H  **TNM stage**  III/IV  I/II  ***SF3B2***  High  Low | 2.037 0.006  (1.224-3.390)  1.00  0.895 0.576  (0.608-1.319)  1.00  0.669 0.121  (0.402-1.111)  1.00  0.824 0.515  (0.461-1.474)  1.00  4.023 0.002  (1.637-9.888)  1.00  1.543 0.032  (1.039-2.292)  1.00 |  | 2.176 0.003  (1.297-3.652)  1.00  1.024 0.905  (0.690-1.520)  1.00  0.618 0.068  (0.369-1.036)  1.00  0.666 0.181  (0.367-1.209)  1.00  3.907 0.003  (1.588-9.610)  1.00  1.600 0.022  (1.071-2.388)  1.00 |

MSI, microsatellite instability; MSS, microsatellite-stable; MSI-L, low level microsatellite instability; MSI-H, high level microsatellite instability. The TNM Staging System is based on the extent of the tumor (T), the extent of spread to the lymph nodes (N), and the presence of metastasis (M). CI, confidence interval; HR, hazard ratio. HRs and their 95% CIs were computed by using Cox hazard proportional regression model.

**Supplementary Table 5.** Alternative splicing analysis of RNA-seq data from SW480 siControl and siRNF6

| **Splicing event** | **Genes (FDR<0.05)** |
| --- | --- |
| Skipped exon | HMGN1, LMNA |
| Mutually exclusive exon | GAPDH, RABGEF1, RP1-309K20.6 |
| Alternative 5' splice site | BCL2L1, FGFR2 |
| Alternative 3' splice site | - |
| Retained intron | AP1G2, CDK11A, CDK5RAP3 (2), CIRBP, CLCN2, DCAF15, DDX11, DDX17, DDX39B, DVL3, EWSR1, FBXL6, GTPBP2, IRF3, ITGA7, KRTCAP2, LMNTD2, LRRC41, POMT1 (2), RELL2, RHOT2, RPL12, SEMA3B, SF3B1, SLC17A9, SLC52A3, SGSM2, SMARCD3, STK19, SYTL1, TMEM259, TRA2A, TSTA3, TTC31, WASH5P, WDR90 |

**Supplementary Table 6.** IC_50_ values for normal and colon cancer cells treated with Pladienolide B 72h

| **Cell lines** | **IC_50_ (nM)** | **95% CI (nM)** |
| --- | --- | --- |
| NCM460 | 17.61 | 10.36-29.92 |
| HT29 | 13.87 | 7.06-27.25 |
| HCT116 | 10.63 | 6.45-17.54 |
| SW480 | 4.86 | 2.65-8.92 |
| DLD-1 | 3.29 | 2.01-5.40 |

CI, confidence interval; IC_50_, half maximal (50%) inhibitory concentration.
