# Supplementary material for: RING-finger protein 6 promotes colorectal tumorigenesis by transcriptionally activating SF3B2
Source: Oncogene. 2021 Oct 5;40(47):6513–26. doi: 10.1038/s41388-021-01872-9 (PMC8616760; doi:10.1038/s41388-021-01872-9)
Supplement: Supplementary file 2 — Supplementary Methods [file 41388_2021_1872_MOESM2_ESM.docx]

**Supplementary methods**

**Immunofluorescence staining**

DLD-1 and SW480 cells were seeded on coverslips in a 6-well plate and fixed with 4% paraformaldehyde and permeabilized with 0.5% Triton X-100. The fixed cells were then blocked with 5% BSA/PBS, and stained with anti-RNF6 antibody (1:80 dilution, 20437-1-AP, Proteintech) overnight at 4°C. Following the incubation with IgG secondary antibody conjugated with Alexa Fluor 488 (1:400 dilution, Yeasen) in the dark for 1 hour, cells were preserved with ProLong Gold Antifade Mountant with DAPI (Life Technologies). Images were subsequently captured by confocal laser scanning microscope.

**Lentivirus infection, small interfering RNA (siRNA)**

For RNF6 or SF3B2 overexpression, CRC cells were infected for 24 hours with recombinant lentivirus encoding human RNF6 (vigene Bioscience, Shandong, China) or SF3B2 (Hanbio Biotechnology, Shanghai, China). Meanwhile, the cells were infected with empty vector lentivirus as a control. Stable transfections were selected for 2 weeks with puromycin (Sigma). To overexpress RNF6 in organoid model, organoids digested into single-cell suspension and infected with lentiviral encoding human RNF6 for 2 hours under centrifugation (500g) and 6 hours incubation at 37℃, followed by reconstitution in Matrigel. Knockdown of RNF6 expression in SW480 was performed by a siRNA targeting RNF6. SW480 was transfected with 50 nM RNF6 siRNA (siRNF6: 5’-GCAAGAUAGAGAACGAGAATT-3’) (Invitrogen) or control siRNA (siControl: 5’-UUC UCC GAACGU GUC ACG UTT 3’) (Invitrogen) using lipofectamine 2000 (Invitrogen). After transfection for 48 hours, the cells were ready for further experiments.

**Cell viability and colony formation assays**

Cell viability of CRC cells was examined by the MTT (5 mg/ml; Promega, Madison, WI, USA). Cell viability of CRC PDOs was measured by CellTiter-Blue cell viability assay (Promega, G8080). For colony formation, cells were seeded on 6-well plates for 7-10 days, and stained with 0.5% crystal violet solution. Colony with >50 cells per colony was counted. All experiments were conducted three times in triplicate.

**RNA extraction and real-time PCR analysis**

Total RNA was extracted from cells and tissues using TRIzol Reagent (Invitrogen). Reverse transcription (RT) was conducted using the PrimeScript^TM^ RT reagent kit (TaKaRa, Japan). For quantitative PCR analysis, aliquots of cDNA were amplified using SYBR Premix Ex Taq^TM^ II (Takara, Japan) on 7500HT Fast Real-Time PCR System (Thermo Fisher Scientific). Primer sequences are listed in **Supplementary Table 1**. Each sample was tested in triplicate. Target gene expression was normalized to β-actin expression. 2-delt delt CT method was employed to evaluate the fold change in gene expression level. 2-delt CT method was employed to determine the relative expression levels of corresponding genes.

**Migration and invasion assays**

For migration and invasion assays, transwell chambers (Corning) and matrigel-coated chambers (BD Bioscience) were used. Cells (2×10^5^/well) were seeded onto the upper chamber in serum-free DMEM. Complete culture medium (supplemented with 10% FBS) was added to the lower chamber as a chemoattractant. After 24-48 hours, cells that have migrated or invaded through the membrane were stained with 0.5% crystal violet and counted. Experiments were conducted in triplicate.

**RNA sequencing**

SW480 cells transfected with siRNF6 or siControl were harvested for total RNA extraction. RNA sequencing and analysis were performed by Beijing Novogene Technology Co. Ltd.

**KEGG pathway enrichment analysis of DEGs**

To generally describe the biological characteristics and functional annotation of candidate DEGs from RNA sequencing, KEGG pathway analysis was performed online. For systematic analysis of DEGs functions, we used the online tool DAVID (https://david.ncifcrf.gov/) to obtain the enriched KEGG terms.

**Analysis of Alternative Splicing (AS) Events**

To identify the AS events from RNA sequencing data, rMATS software was used for quantitative and differential analysis of AS events. Based on the comparison of genes and genomes, rMATS automatically detected and analyzed AS events corresponding to major types of AS patterns. Each AS event has two isoforms: Exon Inclusion Isoform and Exon Skipping Isoform. The quantification of the two isoforms was calculated and divided by the effective length to obtain the corrected expression quantity. Then the ratio of the expression of Exon Inclusion isoforms in the two isoforms was calculated. IncLevel 1 and IncLevel 2 indicate the ratio of siRNF6 and siControl group. Finally, the difference was analyzed. Our threshold for screening for significant AS events is false discovery rate (FDR) less than 0.05.

**Immunohistochemistry (IHC) analysis and tissue microarray assay (TMA)**

Paraffin-embedded colon tissue samples were used. For slides from mice, RNF6, Ki-67 and PCNA signal was assessed by anti-RNF6 antibody (1:100, Invitrogen), anti-Ki-67 antibody (1:500, Abcam) and anti-PCNA antibody (1:2000, Cell Signaling Technology). The proliferation index was determined by counting the numbers of positive staining cells of Ki-67 or PCNA as percentages of the total number of colon cells. At least 1000 cells were counted each time. CRC TMA slides were provided by the Department of Anatomical and Cellular Pathology, the Chinese University of Hong Kong. For slides from patients and tissue microarrays of CRC tissues were stained with SF3B2 antibody (1:100, proteintech). The positive percentage was scored as follows: 0, negative; 1, 1-25%; 2, 26-50%; 3, 51-75%; 4, >75%. The staining intensity was scored as follows: 0, negative; 1, weak; 2, moderate; and 3, high intensity. The staining score of SF3B2 nuclear staining was the product of proportion and intensity scores, ranging from 0 to 12. The high expression of SF3B2 was defined as IHC score >4. The results were scored independently by two pathologists.

**TUNEL staining**

Paraffin slides from AOM-induced WT and *Rnf6* tg mice were used. TUNEL signal was assessed using the DeadEnd^TM^ Colorimetric TUNEL System (Promega), according to the manufacturer's protocol. The apoptosis index was calculated as the percentage of TUNEL-positive nuclei after counting at least 1000 cells.

**Protein extraction and Western blotting**

Total protein from cell lines and tissues was extracted using CytoBuster protein extraction reagent (Merch Chemicals, Nottingham, UK) and protein concentration was measured by the DC protein assay method of Bradford (Bio-Rad, Hercules, CA). Proteins were separated on SDS-polyacrylamide gel electrophoresis (SDS-PAGE) and transferred onto Polyvinylidene difluoride membranes (GE Healthcare, Chicago, IL). The membrane was incubated with primary antibodies overnight at 4°C and then with secondary antibody at room temperature for 1 hour. Proteins of interest were visualized using ECL Plus Western blotting Detection Reagents (GE Healthcare). The antibodies used and their dilutions were listed in **Supplementary Table 2**.

**Dual-luciferase reporter assay**

The HCT116 and HT29 cells were stably transfected with pCMV-RNF6 or pCMV-vector (1 × 10^5^ cells per well) in 24-well plates and co-transfected with luciferase reporter plasmid (200 ng per well) and pRL-TK control vector (50 ng per well) using lipofectamine 2000 (Invitrogen). pGL3-SF3B2 contains the promoter upstream region (2.0 kb) from the transcriptional start site of human *SF3B2*. Cell lysates were harvested at 48 hours post-transfection and luciferase activity was analyzed by the dual-luciferase reporter assay system (Promega, Madison, WI). The experiments were conducted three times in triplicates.

**Co-immunoprecipitation (Co-IP) assay**

Total protein from DLD-1 and SW480 cells was extracted in RIPA buffer containing proteinase inhibitor (Novagen). Immunoprecipitation was performed using RNF6 antibody or IgG. The antibodies were then pulled down with 25 μl protein A/G magnetic beads (Thermo Fisher Scientific Inc, Rockford, IL) for 4 hours at 4°C. After extensive washing, proteins were eluted with low-pH buffer and separated by SDS-PAGE gel, followed by western blotting. The lysate (1% input) was used as a control. The antibodies used are listed in **Supplementary Table 2**.

**Hematoxylin and eosin (H&E) staining**

Mice tissues were embedded in paraffin and sliced in 5 µm sections. Then the slides were stained with H&E following standard protocols and reviewed in a blinded manner by two experienced pathologists.
