# Supplementary material for: RING-finger protein 6 promotes colorectal tumorigenesis by transcriptionally activating SF3B2
Source: Oncogene. 2021 Oct 5;40(47):6513–26. doi: 10.1038/s41388-021-01872-9 (PMC8616760; doi:10.1038/s41388-021-01872-9)
Supplement: Supplementary file 3 — Supplementary Figures [file 41388_2021_1872_MOESM3_ESM.pdf]

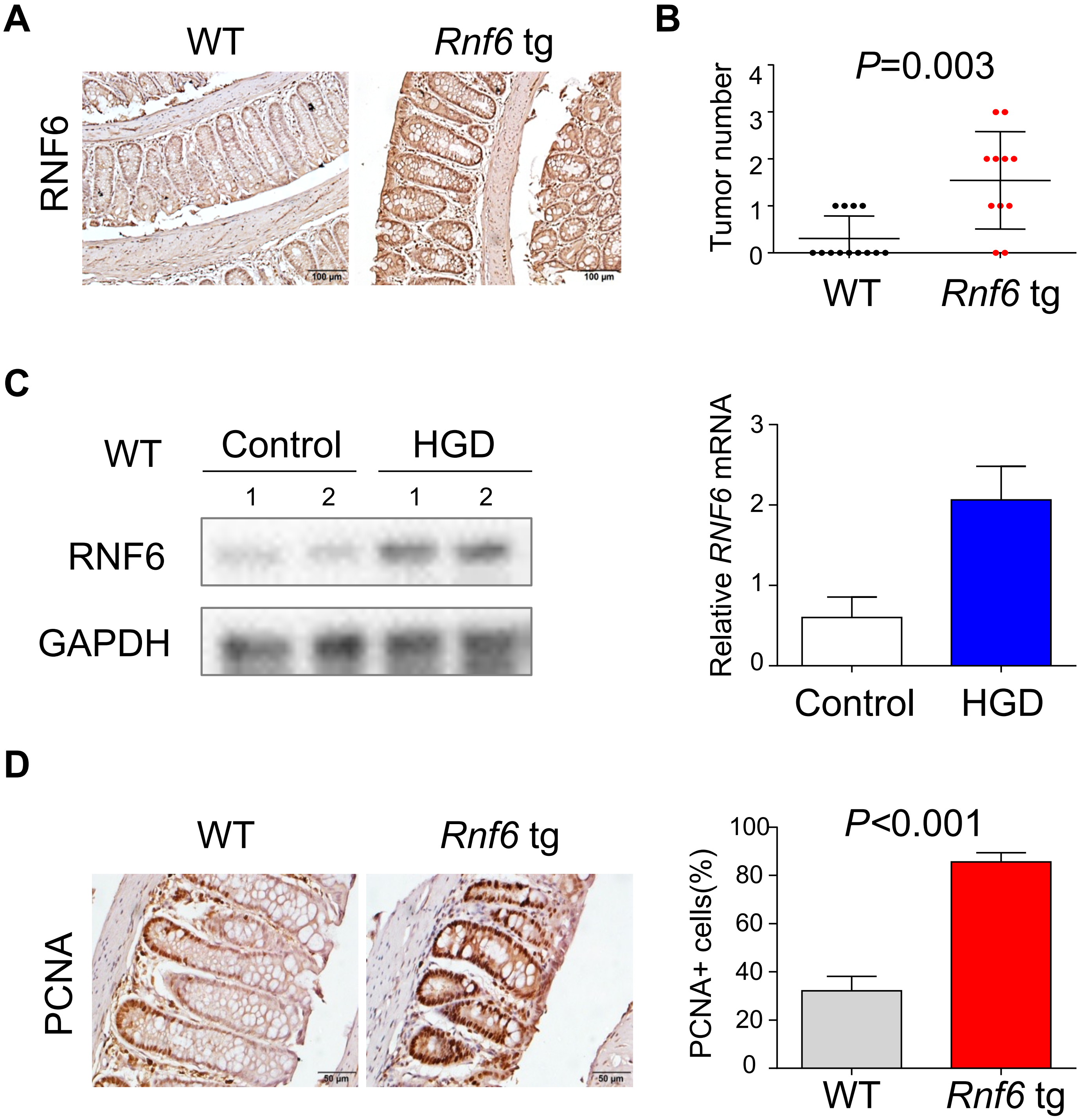

Supplementary Figure 1. Rnf6 tg expression in mice accelerates AOM-induced CRC. **(A)** Representative images of immunohistochemical staining from WT and *Rnf6* tg mouse colons. **(B)** Statistical analysis of tumor number in WT and *Rnf6* tg mice. **(C)** Western blot and RT-qPCR confirmed overexpression of RNF6 in colon high grade dysplasia (HGD) group (n=2) as compared to those without dysplasia (n=9) in AOM-treated wildtype mice. GAPDH was used as a loading control. **(D)** PCNA staining of cell proliferation in WT and *Rnf6* tg mice. Data are expressed as mean ± SD.

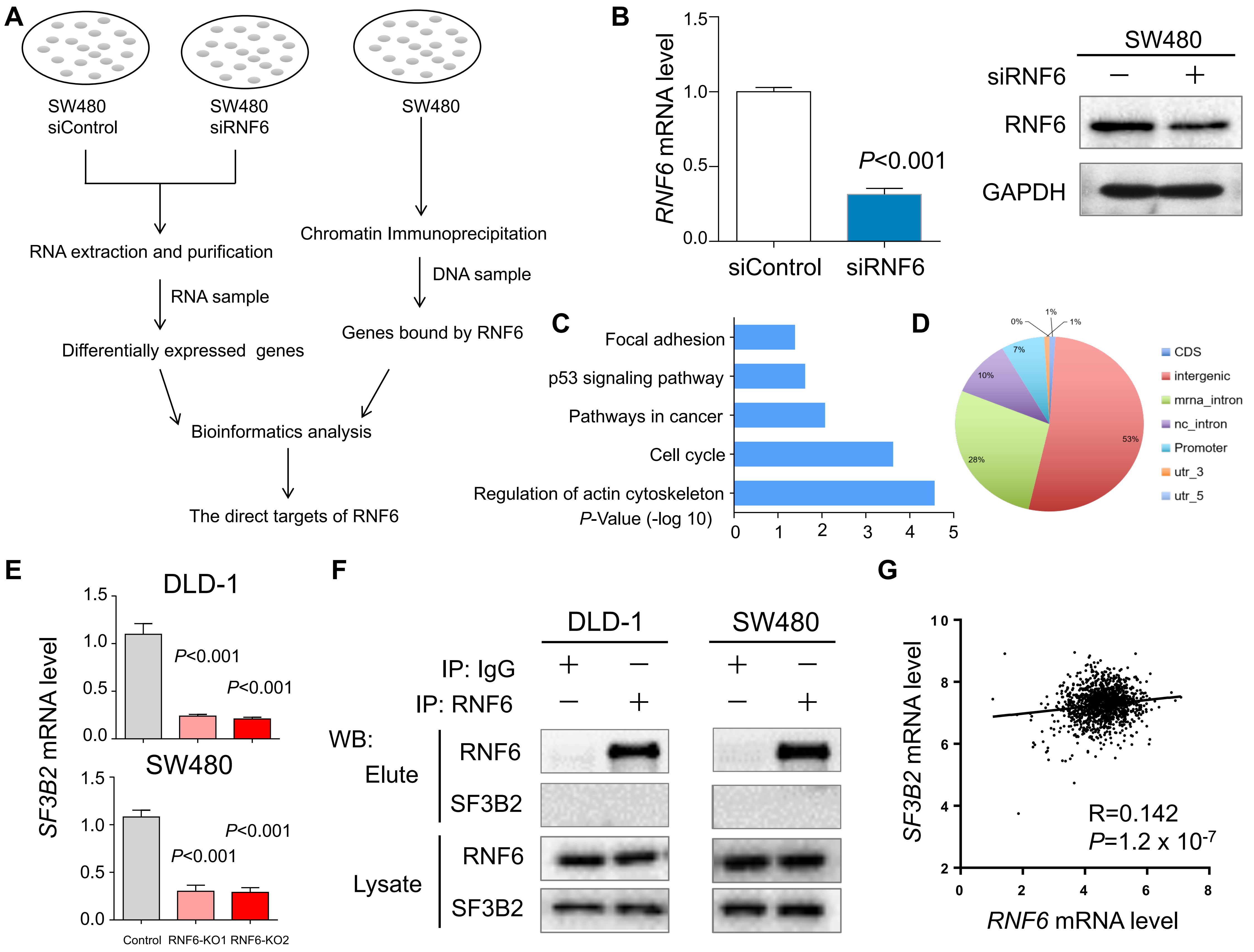

Supplementary Figure 2. Identification of direct transcriptional targets for RNF6. **(A)** Workflow for the identification of RNF6 transcriptional targets. **(B)** RNF6 knockdown in SW480 cells was confirmed by qPCR and Western blot analysis. **(C)** KEGG pathway analysis of differential expressed genes from RNA-seq analysis of SW480 siControl and siRNF6 cells. **(D)** Pie chart of the distribution of RNF6 binding sites from ChIP-Seq. **(E)** SF3B2 mRNA expression upon RNF6 knockout in CRC cells by qPCR (n=3, performed in triplicate). **(F)** Interaction between RNF6 and SF3B2 was examined by co-immunoprecipitation (IP) in DLD-1 and SW480 cells. **(G)** Correlation between RNF6 and SF3B2 mRNA expression in 1,375 human cancer cell lines. Data are expressed as mean ± SD.

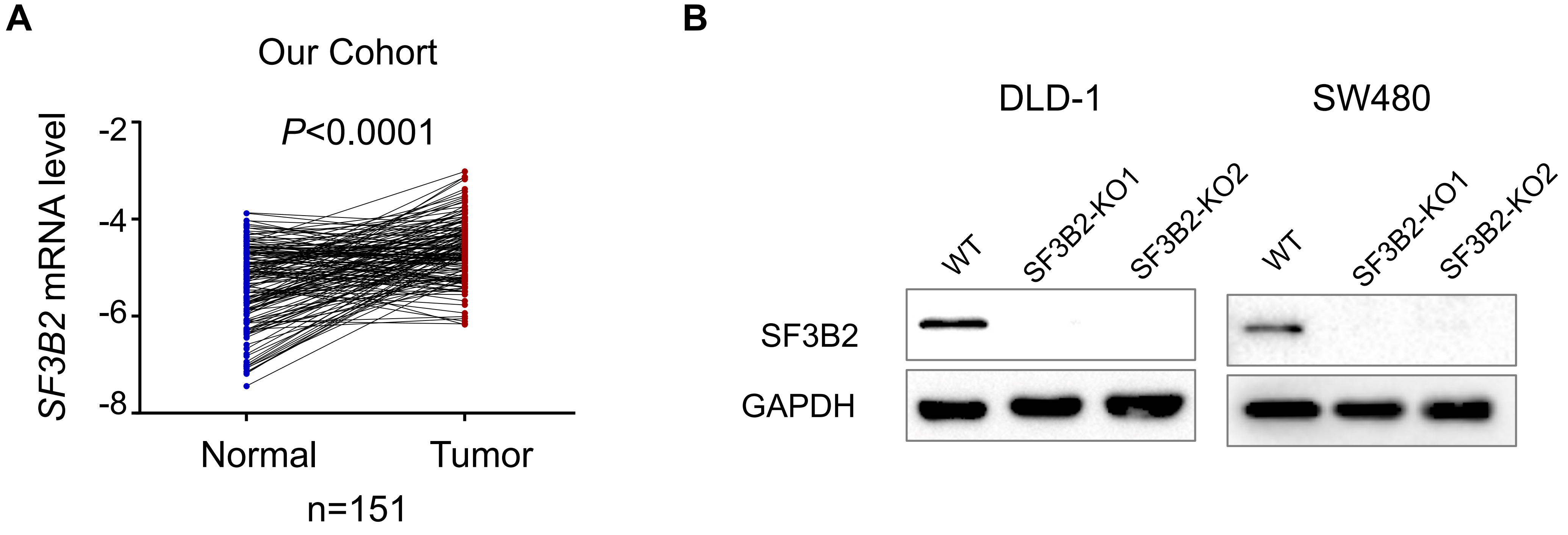

Supplementary Figure 3. SF3B2 functions as an oncogene in CRC. **(A)** Relative SF3B2 mRNA expression in paired CRC tissues of our cohort was assessed by qPCR. SF3B2 mRNA expression was up-regulated in CRC tissues compared to their adjacent non-tumor tissues. **(B)** SF3B2 protein levels in SF3B2 knockout DLD-1 and SW480 were determined by Western blot. Data are expressed as mean ± SD.

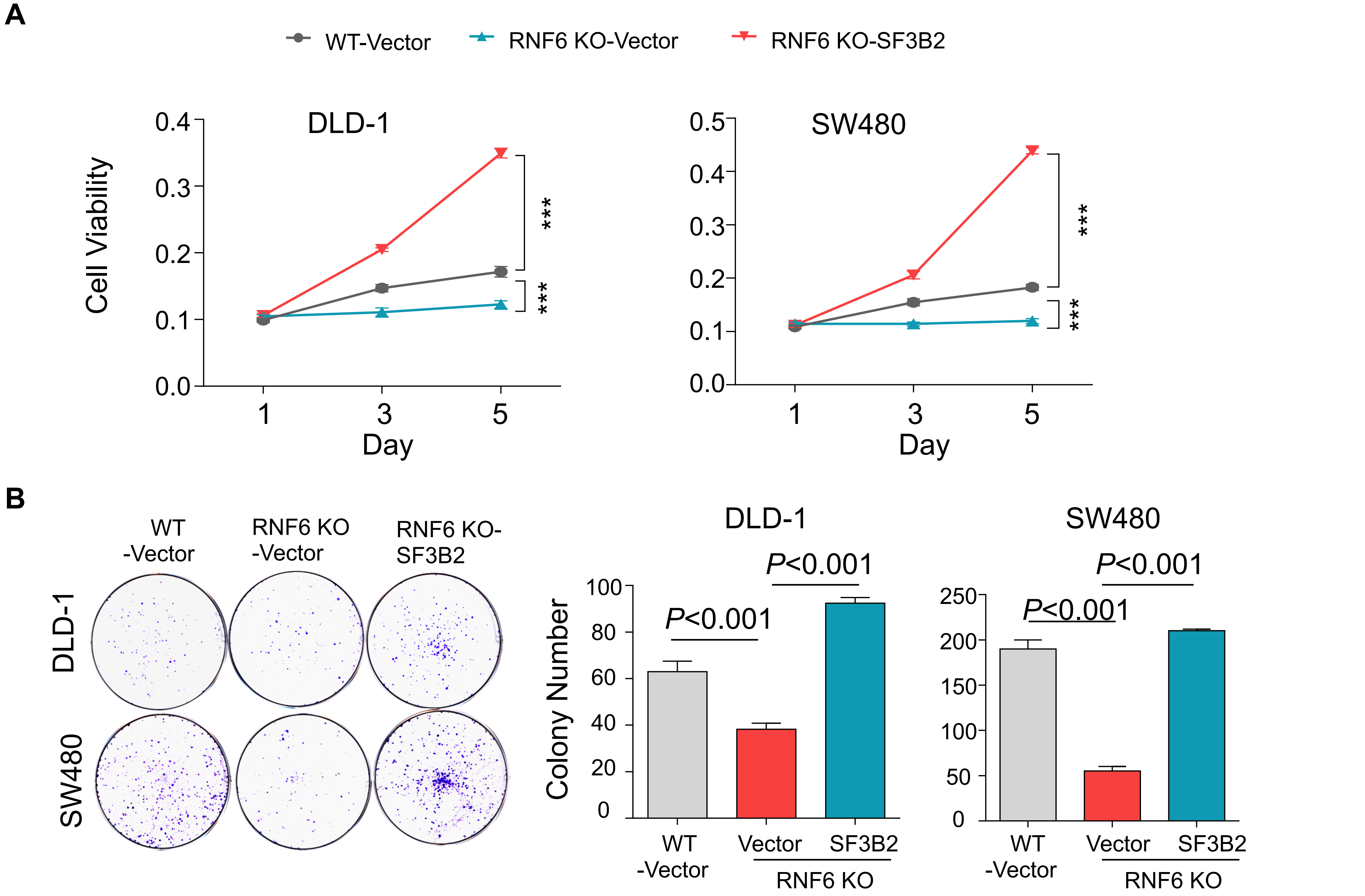

Supplementary Figure 4. Ectopic expression of SF3B2 reverses the anti-tumour effects of RNF6 knockout in CRC cells. (**A** and **B**) MTT and colony formation assays (n=3, performed in triplicate) were used to assess the cell viability and colony number after transfection with SF3B2 plasmid in RNF6 knockout DLD-1 and SW480 cells. Data are expressed as mean ± SD. ****P* < 0.001.

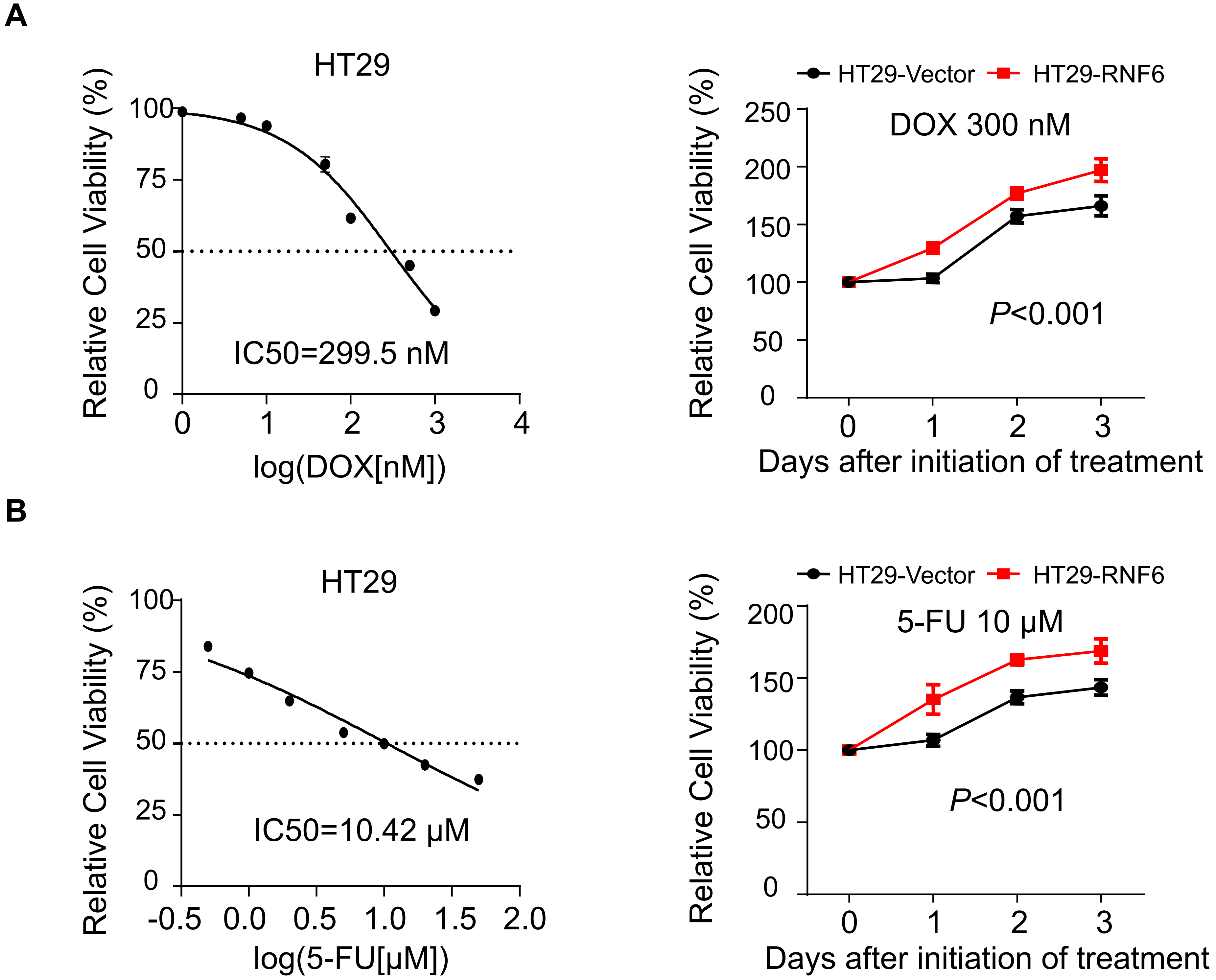

Supplementary Figure 5. Overexpression of RNF6 may confer doxorubicin and 5-FU resistance in CRC. (**A** and **B**) Dose-response curve of doxorubicin (DOX) and 5-FU in wild type HT29 cells for treatment 72h. Drug treatment of doxorubicin (A) or 5-FU (B) in vector-expressed or RNF6 overexpressed HT29 cells. Data are expressed as mean ± SD.
